# Thymoquinone Overcomes Hypoxia-Induced Carboplatin Resistance Through ROS-Independent Apoptosis but Promotes Cancer Stem Cell Enrichment: Implications on Oral Cancer Adaptation and Recurrence

**DOI:** 10.3390/ph18111758

**Published:** 2025-11-18

**Authors:** Ishrat Rahman, Hanan Henidi, Manal M. Alkahtani, Zaha Al Makhlafi, Sahar ElRefai, Manal A. AlSheddi, Rizwan Ali, Sara K. Albassam, Hazar S. Alharbi, Maha G. Omar, Hend M. Salem, Alia Alturki, Hourya Alnofaie, Arwa Alharbi, Nuha Aloraini, Reema Alswied, Samaa Almutairi, Joud Alshahrani, Reem Fahad Alsuwaidan, Shrooq Alqahtani, Aalia Alharthi, Hadeel Alzahrani, Raghad Alkhattabi, Shams A. Altwaim

**Affiliations:** 1 Department of Basic Dental Sciences, College of Dentistry, Princess Nourah bint Abdulrahman University, P.O. Box 84428, Riyadh 11671, Saudi Arabia; imrahman@pnu.edu.sa (I.R.);; 2 Research Department, Natural and Health Sciences Research Center, Princess Nourah bint Abdulrahman University, P.O. Box 84428, Riyadh 11671, Saudi Arabia; hahenidi@pnu.edu.sa (H.H.);; 3 Medical Research Core Facility and Platforms (MRCFP), King Abdullah International Medical Research Center (KAIMRC), National Guard Health Affairs (NGHA), P.O. Box 22490, Riyadh 11426, Saudi Arabia; 4 College of Dentistry, Princess Nourah bint Abdulrahman University, P.O. Box 84428, Riyadh 11671, Saudi Arabia

**Keywords:** oncology, herbal, metabolite, oxygen, chemotherapy, aggressive

## Abstract

**Background:** Carboplatin is a first-line chemotherapy agent for patients with oral squamous cell carcinoma (OSCC), but chemoresistance significantly impacts treatment outcomes. This study evaluated the ability of thymoquinone, a natural metabolite found in food products, to modulate cytotoxicity, ROS, apoptosis, autophagy, and cancer stem cell markers in early- and late-stage OSCC cell models to identify mechanisms of chemoresistance and determine the influence of dietary metabolites on treatment outcomes. **Methods**: OECM-1 cells were treated with concentrations (1 mM to 1 pM) of thymoquinone, carboplatin, or their combination under normoxic and hypoxic conditions. HIF-1α levels were measured using ELISA, and cytotoxicity was assessed by the MTT assay. ROS, apoptosis, autophagy, and cell surface markers (CD44+, CD133+, CD147+) were evaluated. All experiments were repeated three times, and the data were analyzed using GraphPad Prism. Under hypoxia, HIF-1α increased 12-fold. **Results**: Carboplatin demonstrated reduced potency (110 μM) and efficacy (40%) compared to normoxia (82 μM, 88%), accompanied by increased apoptosis (75%) and decreased ROS (25%). Thymoquinone was more potent than carboplatin, further reducing ROS (50%), increasing apoptosis (95%), and downregulating autophagy, while the proportion of CD133+ expressing cells increased significantly (75%) in the hypoxic model. For the combined treatment across both models, thymoquinones’ efficacy remained high (>90%). Between models, no further change in any parameter was observed, except for apoptosis induction, which increased to 65% (normoxia) and 50% (hypoxia). **Conclusions**: Thymoquinones’ superior efficacy under hypoxic conditions demonstrates ROS-independent cytotoxic mechanisms; however, the enrichment of CD133+ cells raises essential questions about long-term therapeutic outcomes and the risks of tumor recurrence. Natural pharmaceutical metabolites can influence the tumor microenvironment, which is highly implicated in cancer therapeutics and cancer adaptation.

## 1. Introduction

Mouth and oral cancers are the 16th most common types of cancer worldwide [1]. Oral squamous cell carcinoma (OSCC) is frequently diagnosed in some regions of the world and is linked to a high mortality rate [2]. As with most cancers, genetic and epigenetic changes contribute to the onset and development of the disease [3]. Over the past few years, the understanding of etiological factors has increased, along with improvements in the correlation between oral cancer and other unconventional factors, such as diet [4]. The risk of oral squamous cell carcinoma differs across countries and regions. There are many variations in geographic distribution, clinicopathological features, and biological traits associated with known risk factors for malignancy [2,4]. The incidence of OSCC is highly correlated with exposure to specific carcinogens, including tobacco, alcohol, environmental pollutants, smokeless tobacco (Shamma) [5,6], high-risk human papillomavirus infection, and ultraviolet radiation of the lip [7].

Southeast Asians have higher oral cancer rates due to their traditional habit of eating raw chewable tobacco, especially betel quid [8]. The incidence rate in Saudi Arabia is 3.86 in 100,000 [1], and in the Jazan region of Saudi Arabia, over 60% of the cases are diagnosed as OSCC; these higher rates are attributed to the widespread use of Shamma in the area [2,7,9]. It has been documented that approximately 90–95% of all oral malignancies globally are squamous cell carcinoma (SCC) [10], with a peak incidence in the sixth and seventh decades of life [2,7]. The prevalence and related mortality rates depict oral cancer as a global priority problem.

Cis-diamminedichloroplatinum (II), commonly known as cisplatin, is a first-generation platinum-containing alkylating agent, and it is the primary chemotherapeutic agent used in treating OSCC [11,12]. This DNA replication inhibitor causes cell cycle arrest and targets both nuclear and mitochondrial DNA to induce apoptosis [11,13]. When drug resistance occurs, other cytotoxic medications, including alternative platinum-based compounds such as carboplatin and oxaliplatin, may be employed [14,15]. Chemoresistance is a common problem that often leads to treatment failure [11,13]. Conventional chemotherapeutic drugs frequently cause toxic effects on non-cancerous dividing cells due to their non-specific nature. There is a need for improved management strategies in OSCC treatment. Furthermore, cisplatin-based combination therapies with other drugs have received considerable attention for addressing drug resistance and minimizing toxicity [16,17]. These advancements aim to enhance therapeutic efficacy while reducing the adverse effects associated with traditional chemotherapy, ultimately leading to better patient outcomes and quality of life.

Advanced-stage aggressive solid tumors have specific traits that set them apart from non-aggressive types, one of which is the formation of a low-oxygen environment. Hypoxia refers to a state of reduced oxygen levels in tissues. In cancers, the excessive oxygen consumption driven by metabolic needs exceeds the supply, creating a hypoxic microenvironment within the tumor [18]. This triggers a series of events that alter the energy metabolism, primarily shifting from aerobic respiration to anaerobic glycolysis, which makes the tumor cell resilient in low-oxygen environments, allowing it to grow. However, severe hypoxia can result in cell death (necrosis), characterized by a central area of necrosis. The hypoxic microenvironment enhances growth, metastasis, and treatment resistance, resulting in increased morbidity and poor clinical outcomes [19,20]. Hypoxia is associated with an aggressive tumor phenotype and can activate protective autophagic processes in cancer cells [21]. It establishes an ideal environment for the transcription of numerous genes through various mechanisms. Hypoxia-induced factor-1a (HIF-1α), a transcription factor, initiates the transcription of multiple proteins that maintain normal homeostatic processes under hypoxic conditions. In cancer, it can promote angiogenesis, vascularization, and the use of alternative metabolic pathways for substrates [22]. HIF-1α overexpression is observed in many cancer types and may play a crucial role in the continued growth, differentiation, and metastasis of solid tumors [19]. Consequently, HIF-1α expression is regarded as a feature of aggressive or metastatic cancers [21], which is linked to the ability of neoplastic cells to survive in a hostile hypoxic microenvironment [19].

The role of reactive oxygen species (ROS) in cancer metabolism has also gained attention, as these molecules can influence tumor progression and response to therapy. ROS are natural byproducts of oxygen metabolism and exist in multiple forms. The most common ones are superoxide (O_2_*^−^), Hydrogen peroxide (H_2_O_2_), Hydroxyl (*OH), Singlet Oxygen (O_2_), Hydroperoxyl (HOO*), Peroxyl (ROO*), and Alkoxyl (RO*) [23]. Elevated ROS levels are frequently associated with oxidative stress in cancer cells, potentially altering signaling pathways that support survival, growth, and metastasis. While ROS play a role in cell signaling at normal levels, higher concentrations can lead to toxicity and cellular damage [24]. ROS are implicated in carcinogenesis and are detected intracellularly in cancer cells, with levels increasing under hypoxic conditions commonly found in aggressive cancers. ROS activate HIF-1 and the pro-inflammatory transcription factor NF-κB, promoting the expression of genes essential for survival, resistance to apoptosis, and a resistant phenotype through autophagic repair [23,25]. Hypoxia and HIF-1 may also induce cancer cells to differentiate into cancer stem cells (CSC) as a survival mechanism in hypoxic environments [26]. Conversely, ROS levels have been shown to inhibit HIF in cancer cells, leading to tumor cell death. As such, the precise role of ROS in promoting neoplastic growth remains unclear, with its function likely dependent on the specific tissue or cell type in which it is present. Notably, research indicates that plant anti-cancer metabolites can also modulate ROS function [27]. This dual role of ROS highlights the complex interplay between oxidative stress and cellular adaptation in tumor biology, suggesting that therapeutic strategies targeting ROS levels could influence cancer progression and treatment outcomes.

CSCs, also known as tumor-initiating cells, comprise a small population within the tumor mass, contributing to tumor heterogeneity and shorter patient survival [28]. These CSCs are instrumental in features well known to be related to cancer aggressiveness, including invasion, metastasis, chemoresistance, and relapse [28]. CSCs may activate epithelial–mesenchymal transition (EMT), a process that enables epithelial cells to differentiate, lose cell–cell adhesion, and become migratory, thereby gaining increased plasticity and metastatic potential—traits required for invasion and metastasis [29,30]. Cell surface markers, such as CD147, CD44, and CD133, can serve as reliable prognostic biomarkers to predict the progression of oral precancer and cancer and may be valuable drug targets [31]. High CD44 expression is associated with higher rates of lymph node metastasis, chemotherapy resistance, and, consequently, poor survival [32,33,34,35], and CD133 appears to confer chemoresistance to cisplatin [36].

Herbal remedies derived from plants are used as traditional medicines and food supplements. These natural products are currently the subject of extensive research due to their medicinal properties. The use of herbal medicines in cancer treatment is considered a rational approach, given that many current chemotherapeutic drugs, such as vincristine from the periwinkle plant [37] and taxanes from the Pacific yew tree bark [38], have plant origins. Moreover, herbal treatments can help mitigate the side effects of conventional cancer therapies, reducing fatigue and improving overall well-being [39]. Common dietary herbs, such as curcumin and black seeds (*Nigella sativa*), are known for their health benefits [40,41]. Black seeds are produced by an annual flowering plant in the Ranunculaceae family. Their medicinal use dates back 2000 years, and in Islamic tradition, they are regarded as a miraculous remedy for all ailments except death. Consequently, black seeds are commonly consumed in Middle Eastern diets. Thymoquinone (TQ), a major phytometabolite in black seeds, is credited with numerous health benefits and pharmacological properties [42,43]. The cold-pressed Nigella sativa extract has been used in clinical trials demonstrating its anti-inflammatory and antimicrobial effects [44]. TQ exhibits a wide range of beneficial biological and pharmacological effects, including immunomodulatory, antioxidant, hypoglycemic, anti-inflammatory, anti-cancer, cardiovascular, and hepatoprotective properties [45]. Despite extensive research highlighting thymoquinone’s anti-cancer properties across various cancers, a notable gap remains in understanding its effectiveness in conditions that more closely reflect the tumor microenvironment. Many previous studies on thymoquinone in oral and other cancers have been conducted in standard laboratory settings (21% oxygen), which do not accurately mimic the low-oxygen environments typical of solid tumors where treatment resistance often occurs. Furthermore, in contrast, most existing literature attributes thymoquinone’s effects to oxidative stress induced by ROS [46]; the reliability of this mechanism in oxygen-poor tumor areas has not been thoroughly investigated. Importantly, very few studies have directly compared the efficacy and potency of thymoquinone with established chemotherapy drugs under similar microenvironmental conditions, limiting our ability to assess its clinical promise. Additionally, while many studies have examined thymoquinone’s effects on cancer stem cell markers, none have fully explored changes in stem cell populations under low-oxygen conditions after treatment, nor have they considered the implications for long-term treatment success and tumor recurrence.

This study aims to address these critical gaps by investigating the cytotoxic pharmacological profile of Thymoquinone compared to Carboplatin and its potential to enhance Carboplatin’s efficacy in treating oral squamous cell carcinoma under normoxic and hypoxic conditions, modeled as early- and late-stage resistant cancer. We examined the apoptotic effects of thymoquinone, its role in inducing autophagy, its ability to modulate ROS, and the expression of CD44+, CD133+, and CD147+ cancer stem cells in both models.

## 2. Results

### 2.1. HIF-1a Expression

**Figure 1:**
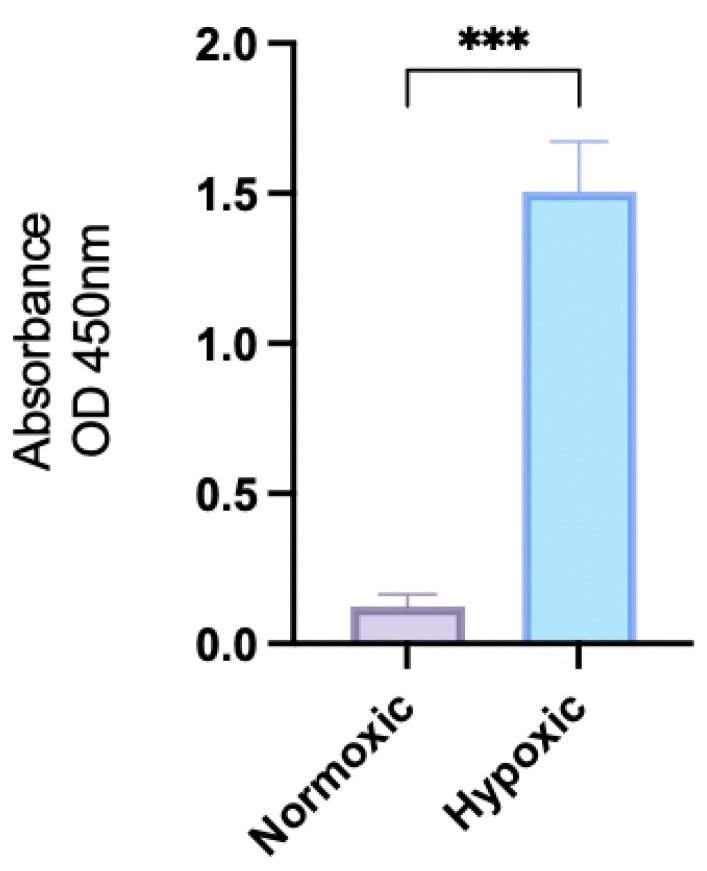
Expression of HIF-1a in OECM-1 cells grown under normoxic and hypoxic growth conditions (*** *p* ≤ 0.001).

ELISA was used, as described in Section 4.3 to assess the influence of depleted oxygen on the OECM-1 cell, HIF-1a expression levels. HIF-1a is a marker to identify aggressiveness and late-stage resistant cancer. The results (Figure 1) show a significant upregulation of HIF-1α (approximately 12×) in OECM-1 cells under hypoxic conditions compared with normoxic conditions. As such, the conditions used for modeling late-stage cancer were approved for use throughout these experiments.

### 2.2. MTT Cytotoxicity Results

**Figure 2:**
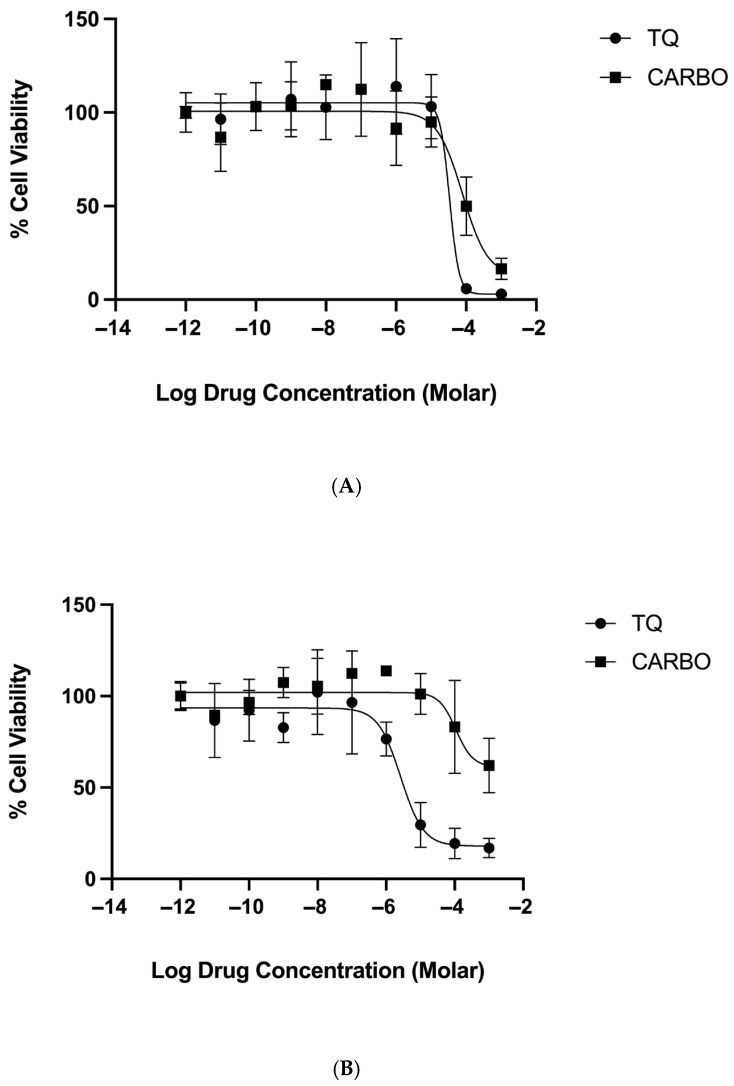
Cytotoxicity of OECM-1 cells with increasing concentrations (1 mM to 1 pM) of thymoquinone (TQ) and carboplatin (CARBO) under normoxic (**A**) and hypoxic (**B**) conditions.

**Figure 3:**
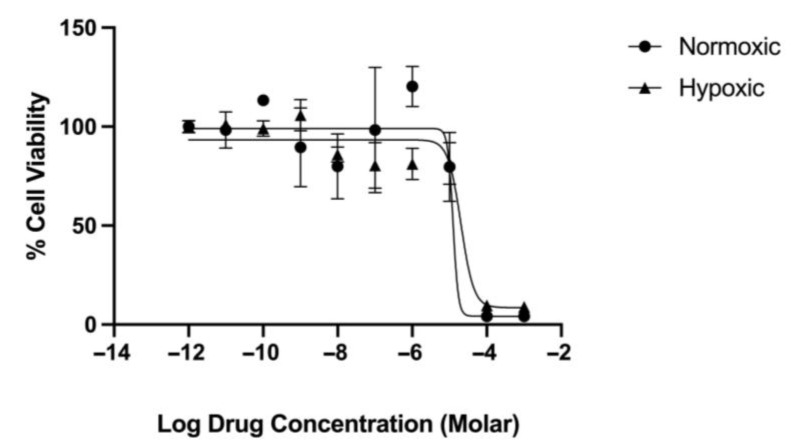
Cytotoxicity of OECM-1 cells with increasing concentrations of thymoquinone (1 mM to 1 pM) combined with a fixed dose of 0.1 mM carboplatin, under normoxic and hypoxic conditions.

The MTT assay is a widely used assay that measures cellular metabolic activity; therefore, it is widely accepted for determining cytotoxic effects in cells. We used the MTT assay as described in Section 4.4 to determine the effect of increasing concentrations of thymoquinone and carboplatin (Figure 2) and a combination of the two drugs (Figure 3) on the cell viability. The span of the curves determines the drug efficacy, and the IC50 identifies the potency values (Table 1).

Results show dose-response curves (Figure 2 and Figure 3) with increasing concentrations of either thymoquinone, carboplatin, or the combination, which caused a reduction in cell viability, thus inducing cytotoxic effects. In normoxic conditions, both thymoquinone and carboplatin have near-equal cytotoxic efficacy (99% and 88%, respectively), although, interestingly, thymoquinone was significantly more potent (2.5× greater) than carboplatin at 28 and 82 µM, respectively. In comparison, under hypoxic conditions, carboplatin showed a marked reduction in efficacy (40%, approximately 50% of the normoxic efficacy); however, thymoquinone maintained a relatively high efficacy, approximately 80%. Furthermore, thymoquinone appears to be more potent at 12 µM under hypoxic conditions, whereas carboplatin exhibits decreased potency at 110 µM under the same conditions.

Results for the combination treatment (Figure 3) show that when carboplatin is combined with thymoquinone, thymoquinone’s efficacy remained above 90% in both normoxic and hypoxic conditions. However, a slight reduction in the thymoquinone potency was noted in the combined treatment, compared to the thymoquinone single treatment for both normoxic and hypoxic conditions, although this difference was not significant.

### 2.3. ROS

**Figure 4:**
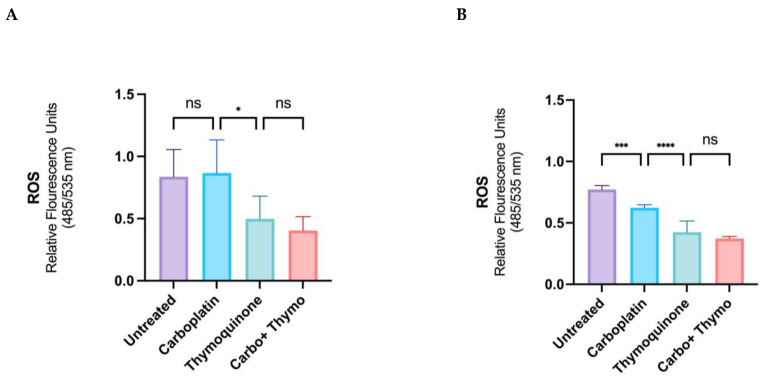
Total ROS levels in live OECM-1 cells after treatment with thymoquinone, carboplatin, carboplatin combined with thymoquinone in normoxic condition (**A**) and hypoxic conditions (**B**) (ns = not significantly different, * *p* ≤ 0.05, *** *p* ≤ 0.001, **** *p* ≤ 0.0001).

The total cellular ROS levels were determined in live cells after treatment with thymoquinone, carboplatin, and the combined treatment, using IC50 concentrations for each agent, as described in Section 4.5 of the methods. This assessment is crucial for understanding the oxidative stress response induced by thymoquinone and carboplatin, as elevated ROS levels can significantly impact cell viability and treatment efficacy. The results (Figure 4) show that under normoxic conditions, carboplatin does not affect the total ROS levels; however, with thymoquinone treatment, there is a significant reduction (approximately 40%) in total cellular ROS, and the reduction is maintained in the combined treatment. Under hypoxic conditions, carboplatin treatment causes a significant reduction in ROS (approximately 25%). Thymoquinone and the combined treatment cause a decrease in total ROS (approximately 50%), similar to the normoxic conditions.

### 2.4. Cell Death and Repair Mechanisms

**Figure 5:**
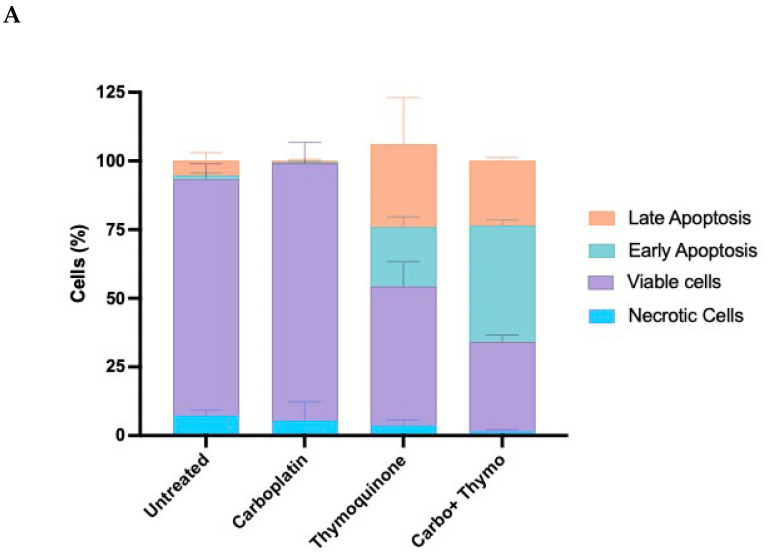

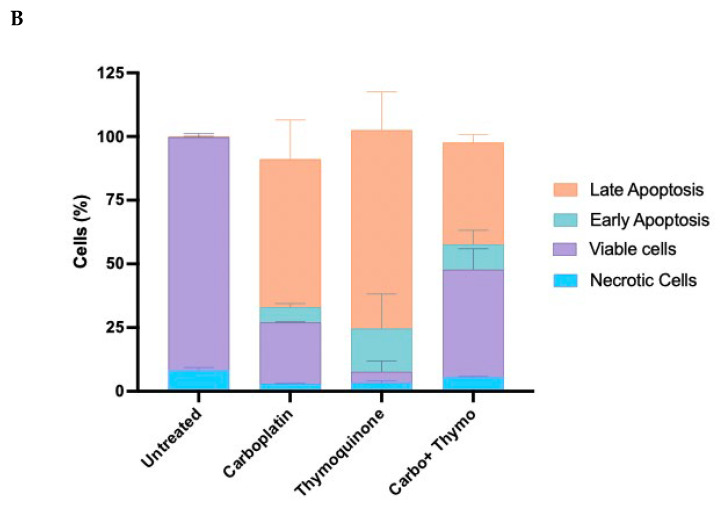
Apoptotic induction of OECM-1 cells on treatment with carboplatin, thymoquinone and thymoquinone combined with carboplatin in normoxic conditions (**A**) and hypoxic conditions (**B**).

The Annexin V marker and PI stain were used during Flow Cytometric analysis, as described in Section 4.6, to determine the percentage of apoptotic cells following treatment with thymoquinone, carboplatin, and both agents combined (Figure 5). The method employed allows for a clear distinction between live, early apoptotic, and late apoptotic or necrotic cells, providing insights into the efficacy and mode of cytotoxic action of the therapeutic agents under investigation. Interestingly, after 24 h of treatment in normoxic cells (Figure 5A), carboplatin did not induce apoptosis; thymoquinone caused approximately 50% of cells to undergo apoptosis, and approximately 65% of cells underwent apoptosis in the combined treatment. Under hypoxic conditions (Figure 5B), thymoquinone treatment induced approximately 95% of cells to undergo apoptosis, a rate significantly higher than under normoxic conditions. Under hypoxic conditions, carboplatin promoted approximately 75% of the cells to undergo apoptosis.

**Figure 6:**
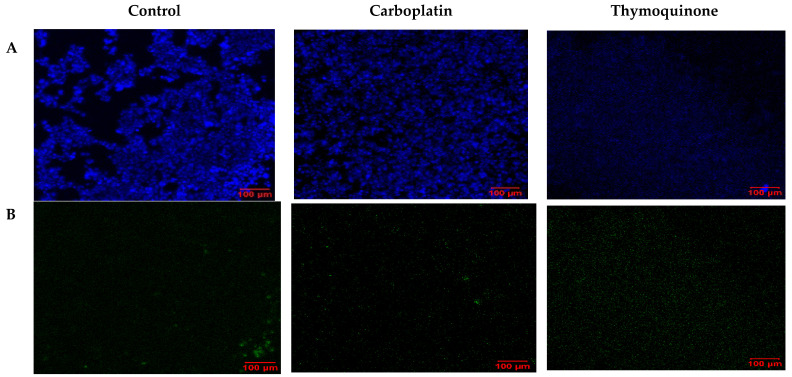

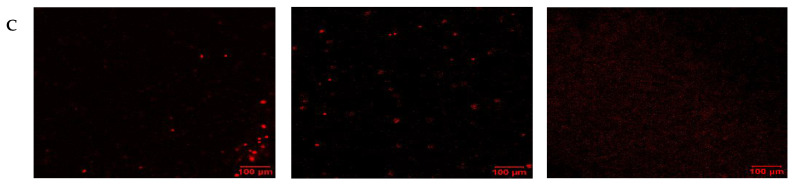
Confocal LSM image of OECM-1 cells on treatment with carboplatin, and thymoquinone in normoxic conditions, with nuclear stain (**A**), apoptotic stain (**B**) and necrotic stain (**C**).

**Figure 7:**
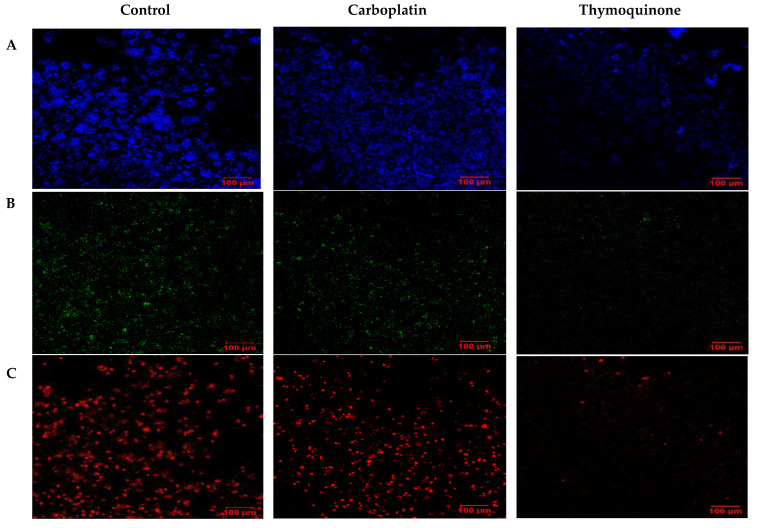
Confocal LSM image of OECM-1 cells on treatment with carboplatin, and thymoquinone in hypoxic conditions, with nuclear stain (**A**), apoptotic stain (**B**) and necrotic stain (**C**).

To visualize the apoptotic cells, confocal laser scanning microscopy (LSM) was employed. In this case, single treatments with thymoquinone and carboplatin were compared with untreated control cells. Under normoxic conditions (Figure 6), carboplatin induced very little apoptosis, as evidenced by minimal green staining. Interestingly, with thymoquinone treatment, an absence of nuclear stain and blue stain bleeding into the media was evident. In comparison, under hypoxic conditions (Figure 7), the control showed higher levels of apoptosis and necrosis, reflecting the effect of the harsh hypoxic conditions on untreated cells. Both with carboplatin and thymoquinone treatment, a relatively greater number of cells were seen to be apoptotic, and more were also necrotic compared with the normoxic. Furthermore, with thymoquinone, not all the cells were lysed, as evidenced by the presence of nuclear stain.

**Figure 8:**
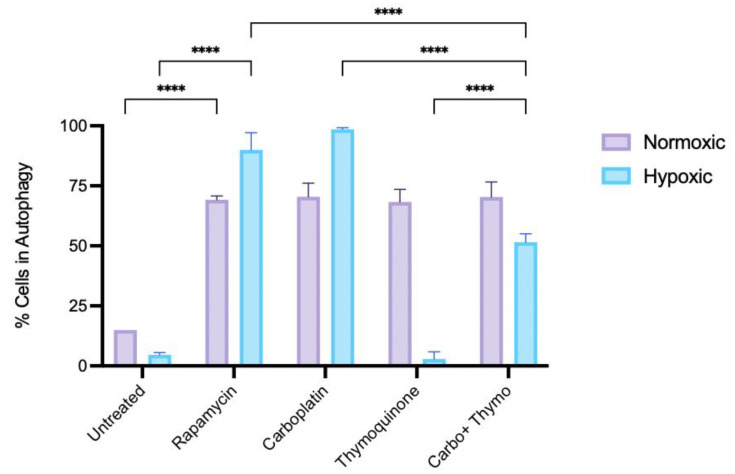
Acridine orange staining of autolysosome, in OECM1 cells in hypoxic and normoxic conditions, under treatment with rapamycin, carboplatin, thymoquinone and thymoquinone combined with carboplatin (**** *p* ≤ 0.0001).

Furthermore, to assess autophagy levels, acridine orange staining was used to detect autolysosomes in control and treated cells. Results (Figure 8) show that untreated cells under normoxic conditions exhibit far less autophagy than cells treated with rapamycin, carboplatin, thymoquinone, or thymoquinone combined with carboplatin, indicating that treatment-induced cytotoxicity in early-stage non-resistant oral cancer triggers autophagy as a mode of cell death or a repair mechanism. Interestingly, in the hypoxic condition, rapamycin and carboplatin further enhance autophagy compared to the normoxic condition; however, thymoquinone treatment causes negligible autophagy.

### 2.5. Phenotyping with Stem Cell Surface Marker Expression

**Figure 9:**
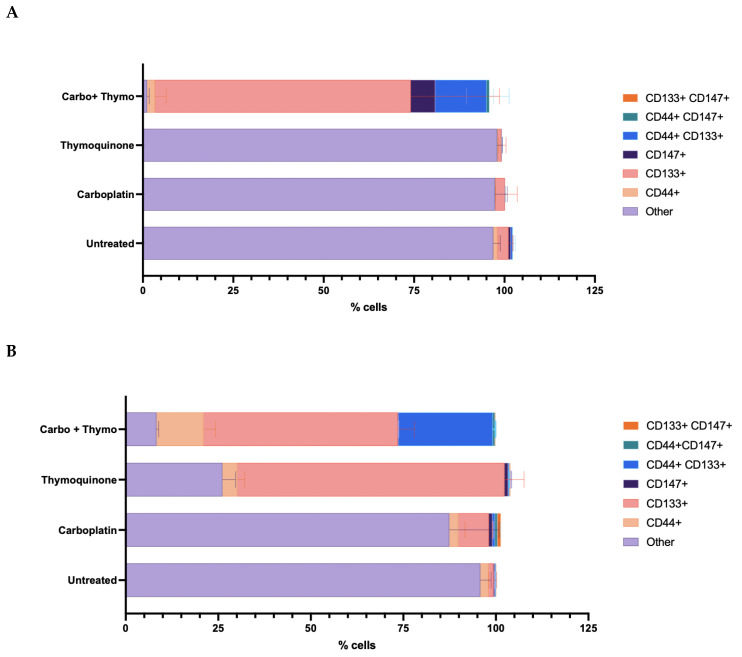
Stem cell surface marker expression was determined treatment of OECM-1 cells with carboplatin, thymoquinone and thymoquinone combined with carboplatin in normoxic conditions (**A**) and hypoxic conditions (**B**).

Stem cell surface marker expression (CD147+, CD133+, and CD44+) was identified in OECM-1 cells treated with control and test agents under both normoxic and hypoxic conditions (Figure 9). Under normoxic conditions (Figure 9A), single treatments with either carboplatin or thymoquinone did not significantly alter the phenotype compared with the untreated control (*p* < 0.05), in which more than 95% of the cells expressed neither of the detected markers. The combination treatment of thymoquinone and carboplatin resulted in a significant increase in CD133+ cells (over 70%) (*p* < 0.0001), a double-positive CD44 + CD133+ phenotype emerged (20–25%), and a small proportion (approximately 5%) of CD147+ cells; however, none of these were significant (*p* > 0.05). Interestingly, under hypoxic conditions (Figure 9B), thymoquinone treatment led to a distinct phenotype compared with normoxia, with approximately 70% of cells expressing CD133+ (*p* < 0.0001). Whereas the phenotype of cells treated with carboplatin was similar to that of normoxic cells, mainly other (85%), with a very small proportion (10%) of CD133+ emerging cells (*p* < 0.05). With combined treatment, a larger proportion of CD44+ cells (approximately 15%) and a larger proportion of double-positive CD44+ CD133+ cells (approximately 25%) emerge relative to the normoxic condition; however, neither was significantly different (*p* < 0.05).

## 3. Discussion

In the current study, we modeled early- and late-stage OSCC using a hypoxic chamber to achieve depleted oxygen levels, enabling us to closely observe cellular responses to various treatments and their implications for therapeutic strategies in the OECM-1 cell line. In vivo studies demonstrate that aggressive tumors often exhibit oxygen levels ranging from 1 to 2% or lower [47], whereas oxygen levels below 0.02% are classified as severe hypoxia [48]. Maintaining an oxygen level of 0.1% is deemed sufficient to avert severe hypoxia, while accommodating the partial pressure of oxygen typically observed in the deeper regions of solid tumors. Our findings underscore the importance of understanding the tumor microenvironment, as hypoxia can significantly impact the effectiveness of treatment regimens and influence the apoptotic pathways activated in cancer cells [46]. As expected, HIF-1α levels were substantially elevated in hypoxic conditions, underscoring its role as a critical regulator of cellular adaptation to low-oxygen environments, which promote cellular survival, aggressiveness, and metastasis [21,49], thereby complicating treatment outcomes.

The elevated HIF-1a levels confirmed that our model for late-stage chemoresistant oral squamous cell carcinoma was suitable for further research. The presence of HIF-1α under low oxygen conditions can promote the expression of anti-apoptotic factors while suppressing pro-apoptotic signals, creating a protective niche for malignant cells [22]. Moreover, targeting these hypoxic regions with therapies aimed at re-establishing normal oxygen levels may sensitize tumors to conventional treatments by restoring their ability to undergo apoptosis [18]. This dual approach, integrating traditional cytotoxic agents like carboplatin with strategies that disrupt the hypoxic response, could pave the way for more effective therapeutic regimens against resistant forms of OSCC [50].

Measuring cellular metabolic activity is often considered a standard method for determining cell viability or cytotoxicity. From the MTT cytotoxicity assay, we characterized the pharmacological efficacy and potency of thymoquinone, comparing it to the first-line chemotherapeutic agent carboplatin. The results from the MTT cytotoxicity assay showed a significant reduction in carboplatin’s efficacy and potency in the hypoxic model compared to the normoxic model. The data highlight that the hypoxic model is chemoresistant to carboplatin. As mentioned earlier, previous literature indicates that HIF-1α expression in oxygen-depleted environments confers chemoresistance in cancers [20]. Furthermore, our data clearly show that thymoquinone is more potent and efficacious than carboplatin in both early- and late-stage chemoresistant OSCC models, and that it is more potent in chemoresistant cells than in non-resistant cells (early-stage model). As such, thymoquinone alone could be a useful therapeutic biomolecule in the treatment of late-stage OSCC. This suggests that the tumor microenvironment plays a crucial role in determining the efficacy of thymoquinone and carboplatin, underscoring the need for tailored therapeutic strategies that account for tumor oxygen levels. The clinical response to cancer therapy varies significantly based on the tumor’s oxygenation status, emphasizing the importance of integrating hypoxia-targeted therapies in cancer treatment protocols [51,52]. Moreover, combination treatment compromised thymoquinone’s potency but did not compromise its efficacy; in fact, a modest enhancement in efficacy was observed in the chemoresistant model. In addition, in the chemoresistant, hypoxic cell model, thymoquinone’s potency was 5 times that of carboplatin when used as a treatment alone. Hence, doubling the dose of thymoquinone could overcome carboplatin’s reduced efficacy. Therefore, in the early stage, OSCC combined therapy may provide little or modest cytotoxic benefit. Although in carboplatin-induced chemoresistant tumors, combination therapy may be useful. Other studies that have shown synergistic effects with various combinations of chemotherapeutic agents [49], the current study highlights differences in combination effects between resistant and non-resistant cell lines. However, further optimization experiments may be required to observe additional benefits of combination treatments and to elucidate the mechanisms underlying resistance. Furthermore, preclinical screening may not reflect clinical outcomes, underscoring the importance of conducting well-designed clinical trials to validate the effectiveness of proposed treatment combinations in real-world settings and to determine whether alternative dosing strategies or treatment schedules could yield improved outcomes. Here, we report that thymoquinone may be considered for future clinical trials as a first-line single chemotherapeutic agent, and that its addition as an adjunct could enhance treatment efficacy in chemoresistant or late-stage cancer, as earlier studies have highlighted thymoquinone’s usefulness in cancer co-therapy [49,53].

The precise role of ROS in cancer growth and death remains unclear, and it may vary by tissue and treatment. Interestingly, studies show that plant anti-cancer compounds can also influence ROS activity [27]. Thymoquinone may enhance the efficacy of conventional therapies by modulating ROS levels to promote cancer cell apoptosis while protecting normal tissues. Although thymoquinone is known to be a powerful antioxidant, a report highlights thymoquinone’s ability to prevent cancer cell growth by inducing ROS production [54]. Thus, understanding the balance of ROS production and elimination is key to developing targeted therapies that enhance patient outcomes. Here, we explored live-cell ROS levels in OSCC OECM-1 cells treated with thymoquinone and carboplatin, aiming to elucidate the single and synergistic effects of these compounds on ROS dynamics and their subsequent impact on cell viability. In normal oxygen conditions, modeled as an early-stage, less aggressive cancer, carboplatin treatment did not affect the ROS levels. However, under hypoxic conditions, carboplatin treatment caused a significant reduction in ROS (approximately 25%). Taken together with the cytotoxic data, which show that carboplatin was more efficacious and potent in normoxic conditions than in hypoxic conditions, the reduction in ROS during carboplatin treatment under hypoxic conditions is not associated with cell death but may instead reflect the activation of resistance mechanisms. Reports highlight that ROS triggers HIF-1α and the pro-inflammatory transcription factor NF-κB, which encourages the expression of genes vital for survival, resistance to cell death, and a resistant phenotype through autophagic repair [23,25]. In our case, the reduction in ROS following carboplatin treatment in the hypoxic model may be linked to autophagic repair, as autophagy appears to be higher in the chemoresistant model on carboplatin treatment (Figure 8), and typically, platinum-containing chemotherapeutic drugs, including carboplatin, are known to be clinically less effective in late-stage OSCC due to chemoresistance. Interestingly, a recent article highlighted the superior ability of a novel platinum agent to induce cancer cell death by initiating an intracellular ROS storm, demonstrating its effectiveness against chemoresistant cancers [55]. This clearly demonstrates ROS generation as a mode of cell death; conversely, reduced ROS may be implicated in chemoresistance [56]. Thymoquinone is an antioxidant that reduces oxidative stress and may prevent oral cancer. It inhibits autophagy in hypoxic, chemoresistant cells, boosting treatment efficacy. Combining thymoquinone with carboplatin decreases autophagy, potentially enhancing therapy and protecting against oxidative damage. Its effects on ROS, autophagy, and apoptosis suggest metabolic reprogramming that contributes to drug resistance. One study reported that thymoquinone reduced NF-κB activity and increased caspase-3 levels in a HepG2 cancer cell line [57], supporting the idea that thymoquinone’s anti-inflammatory effects align with its pro-apoptotic effects. Another more recent study used gene expression profiles in a mouse KB cell line, derived from an oral epidermoid carcinoma, which showed that thymoquinone downregulated the PI3K/AKT/mTOR pathway, in addition to downregulating BCL2 and upregulating BAX and downregulating pro-inflammatory cytokines [58], highlighting thymoquinone’s activity to repress the survival pathway while promoting apoptosis. The present study did not evaluate PI3K/AKT/mTOR; hence, further research is necessary to clarify the mechanisms of cytotoxicity and the proposed mechanisms of resistance. Additionally, it is conceivable that mechanisms of cytotoxicity, apoptosis, adaptive resistance, and signaling, in general, are not only governed by the treatment but also by the cell type, the cellular landscape, and the microenvironment, collectively referred to as the cell texture [59]. However, its anti-cancer properties, mediated by its antioxidants, represent another mode of action [45]. Understanding the balance between these opposing effects is crucial for developing effective therapeutic strategies. Additionally, investigating the molecular pathways involved could provide insights into how thymoquinone can be optimized for cancer treatment while minimizing potential cytotoxicity to normal cells.

Furthermore, the complex interaction between apoptosis and the tumor microenvironment is vital in understanding cancer progression and treatment resistance. Recent studies have shown that a hypoxic environment not only affects cell metabolism but also modulates apoptotic pathways, often leading to increased survival of cancer cells [20]. Surprisingly, under normal oxygen conditions, carboplatin treatment for 24 h did not trigger apoptosis, suggesting that its cytotoxic effect is either independent of apoptosis or causes a delayed apoptotic response. However, under hypoxic conditions, about 70% of the cells underwent apoptosis. The cytotoxicity data suggest that carboplatin is more effective and potent in normoxic cells, indicating that apoptosis is not the main mechanism of cell death under normal oxygen conditions in the OECM-1 cell line. Our findings challenge the traditional view that carboplatin induces cell death primarily through apoptosis [60]. However, apoptosis appears to be the favorable mode of action in the hypoxic, more resistant cell line. In addition, there is an increasing body of evidence suggesting that carboplatin and other platinum-containing agents, such as cisplatin, may induce cytotoxicity via alternative mechanisms [50], including necroptosis, pyroptosis [61], and autophagy-dependent ferroptosis [62]. Our data suggest that autophagy adaptive repair is activated upon treatment, and the heightened levels of autophagy observed with carboplatin treatment in the hypoxic model indicate that autophagy may be a mechanism by which cellular resistance is achieved, as well as a potential mechanism for switching on the more controlled and immune-evading mode of cellular death, apoptosis. Carboplatin is an alkylating agent that is known to cross-link DNA, prevent DNA replication and repair, ultimately causing DNA fragmentation and inducing cellular apoptosis. However, we found that the mechanism of carboplatin’s cytotoxicity differs between early- and late-stage chemoresistant cancers. In our hypoxic model, the reduction in ROS correlates with carboplatin chemoresistance, as well as a preference for apoptosis and heightened autophagic repair. Targeting the ROS pathway, shifting away from apoptosis, and inhibiting cellular repair could enhance carboplatin’s effectiveness in overcoming chemoresistance, potentially improving treatment outcomes for patients with late-stage cancers [63]. However, further research is essential to provide mechanistic evidence for thymoquinones’ ability to induce ROS-independent apoptosis and to understand how these mechanisms could be leveraged to sensitize resistant cancer cells to carboplatin therapy, paving the way for novel combination treatment strategies.

The confocal data showed that thymoquinone caused a strong, robust cytotoxic effect in the early-stage model, lysing cells and allowing the stain to bleed into the media. In contrast, in the late-stage model, not all cells were lysed, indicating that hypoxic conditions rendered cells more resistant to treatment. Under hypoxic conditions, all treatments induced a greater proportion of cells to undergo apoptosis, compared with the normoxic condition. Indicating a preference of the cancer cells to switch to a more controlled state of programmed cell death, under a hypoxic environment. This may be in an effort to conserve cells that are becoming resistant and regulate the microenvironment such that the immune system is less provoked. With processes of cell death such as necroptosis and pyroptosis, the immune system is provoked, leading to a greater inflammatory response [61]. In contrast, apoptosis is a process by which the immune system is not heavily provoked; as such, cancer cells may favor this pathway to evade detection and destruction by immune surveillance, allowing them to persist and adapt in challenging environments. This strategic preference for apoptosis not only aids in their survival but also contributes to tumor progression, ultimately complicating treatment approaches and necessitating the exploration of therapies that can effectively target these evasion mechanisms. A recent study highlighted the ability of plant metabolites to phosphorylate and activate the AKT/mTOR pathway, which is associated with survival and resistance, while simultaneously inducing apoptosis in breast cancer cells [64]. Furthermore, hyperactivation of mTOR has been associated with cisplatin chemoresistance [65]. Consequently, understanding the intricate balance between these forms of cell death is crucial for developing innovative therapeutic strategies that can disrupt cancer cells’ ability to exploit apoptosis and enhance their vulnerability to immune-mediated destruction. In the scenario discussed above, the detection of apoptosis may indicate cellular reprogramming to promote resistance to conventional therapies, highlighting the importance of identifying biomarkers that can predict treatment outcomes.

Moreover, low oxygen and HIF-1α can cause cancer cells to transform into cancer stem cells (CSCs) as a survival strategy in low-oxygen settings [26]. CD44 and CD133 are common markers for identifying cancer stem cells, and high expression levels of these markers are associated with a poor prognosis and increased recurrence [32,36]. CD147, a glycoprotein, promotes glucose metabolism, is involved in the epithelial–mesenchymal transition and metastatic migration through the PI3K/AKT pathway, and is therefore associated with a poor prognosis. However, unexpectedly, the CSC marker expression profiles of untreated cells in both hypoxic and normoxic environments did not differ, as most cells did not express either marker. Thus, these CSC markers were not implicated in aggressiveness or carboplatin chemoresistance in the OECM-1 cell line. However, thymoquinone treatment polarized aggressive late-stage cancer cells toward the CD133+ or CD44 + CD133+ phenotype, which strikingly correlated with decreased autophagy in the hypoxic model, suggesting that these markers may play a role in thymoquinone-mediated cytotoxic responses, especially in aggressive cancer cells. The CD133+ enrichment observed under thymoquinone treatment reflects selection pressure rather than a differentiation event, as the cell population adapts to a more resistant rather than a specialized phenotype. It is essential to note that the cellular characteristics of these cells are determined by the levels of CD44+ and CD133+ [66]. Typically, the emergence of CD133+ cells, and more so the double-positive CD44+CD133+ phenotype, correlates with a higher proliferative capacity and inferred chemoresistance. However, our results are in stark contrast with the existing literature. Additionally, elevated levels of CD44+ are known to induce a hypoimmunogenic state, thereby allowing immune evasion [67]. In contrast, reports indicate that CD44+ may be associated with immune cell responses and PD-L1 expression in lung adenocarcinoma [68]. Furthermore, CD133+ cells are known to modulate autophagy and apoptosis [69]. The current study finds that thymoquinone treatment, which maintains its cytotoxicity against aggressive OSCC, is associated with increased CD133+ expression, lower ROS levels, reduced autophagy, and increased apoptosis. This highlights the crucial role of the cancer microenvironment in shaping cancer cell behavior and influencing treatment outcomes. Although the current study found that thymoquinone retains cytotoxicity in the resistant phenotype, reports suggest that plant metabolites can activate mechanisms of resistance in bacteria and cancer [59,64,70]. The expression of the CD133+ CSC marker in the aggressive phenotype may reflect a change in cell behavior that facilitates survival, suggesting that cancer cells are dynamically responding to treatment pressure. The identification of CSC markers within a tumor indicates that these cells may be able to adapt to therapeutic challenges. Consequently, even when the majority of the tumor mass is diminished or obliterated, the residual CSCs could initiate tumorigenic growth and increase the likelihood of relapse. Therefore, it is imperative to determine whether the existing in vitro observations of CSC enrichment are consistent with findings within in vivo and clinical settings. In the clinical setting, thymoquinone is mostly consumed through the diet. Hence, understanding how dietary factors affect treatment efficacy and patient responses is vital for improving therapies and making personalized, informed dietary recommendations during treatment. This intricate interplay may further complicate treatment outcomes by introducing variables that could influence both tumor dynamics and patient metabolism. Examining the link between diet and treatment response could yield important insights into improving cancer outcomes. Furthermore, the potency and efficacy of thymoquinone are crucial determinants, delivering rapid cytotoxic action that prevents cancer cells from completing their adaptive survival response is a new concept in treatment strategies; treatments must act faster than cancer cells can adapt to them. This offers a potential strategy for overcoming chemoresistance by considering treatment kinetics. Hence, it is essential to explore further mechanisms that shed light on the adaptation in different tumor microenvironments and their impact on treatment responses, potentially guiding the development of more effective therapeutic strategies that target both the bulk tumor and its resilient stem cell population. Understanding these dynamics could lead to personalized treatment approaches that improve patient outcomes in chemoresistant cancers.

## 4. Materials and Methods

### 4.1. Cell Culture and Treatment with Test and Control Drugs

OECM-1 cells (SCC180, Merck, Darmstadt, Germany) were cultured in a T75 flask using RPMI-1640 supplemented with 10% FBS and 5% pen-strep, maintained at 20% O_2_ and 5% CO_2_ at 37 °C. They were passaged at 80–85% confluence with a split ratio of 1:3 to 1:6. Cells were dislodged with 10 mL PBS plus 4 mM EDTA, centrifuged at 800 rpm for 10 min, and resuspended in 10 mL of supplemented RPMI. Cells were seeded into 96- or 6-well plates and allowed to grow overnight before treatment. Treatments included Thymoquinone, Carboplatin (positive control), Combination drugs, and Phosphate-buffered saline (PBS) as a negative control. Single-concentration treatments used the IC50 value derived from the MTT cytotoxicity assay (described in Section 4.4). Cells were incubated for 24 to 48 h, depending on the endpoint assay, under normoxic conditions (humidified atmosphere with 20% O_2_ and 5% CO_2_ at 37 °C), oxygen-depleted conditions [45,46], and hypoxic conditions (humidified atmosphere with 0.1% O_2_ and 15% CO_2_ at 37 °C) using the MGC AnaeroPack System.

### 4.2. Cell Lysis and Protein Determination

Cells were seeded in 6-well plates and treated with drugs at IC50 concentrations, as described in Section 4.1. After 48 h under normoxic and hypoxic conditions, cells were detached with PBS + 4 mM EDTA, collected, centrifuged, and resuspended in PBS. They were then lysed with Qiagen Cell Lysis buffer (Qiagen, Hilden, Germany). Protein concentrations were measured using the BCA assay, following the manufacturer’s instructions. Lysates were stored at − 90 °C until use.

### 4.3. HIF-1a ELISA

ELISA was conducted using a HIF-1a Human ELISA kit (ab171577, Abcam, Cambridge, UK) on OECM-1 cell lysates (1 mg/mL) from untreated cells (basal) in normoxic and hypoxic conditions for 36 h. The protocol was performed following the manufacturer’s instructions. Colorimetric analysis was performed for the wells, measuring the absorbance at 450 nm.

### 4.4. MTT Cytotoxicity Assay

Serial dilutions of thymoquinone, curcumin, and cisplatin (1 mM to 1 pM) were prepared in PBS. Cells were seeded at 5000 per well in RPMI with antibiotics and 10% FBS in 96-well plates, then incubated overnight in 5% CO_2_. Treatments included 100 μL of each dilution, alone or combined with 100 μM carboplatin, and were applied to cells in either oxygenated or hypoxic conditions for 48 h. The MTT assay involved adding 10 μL of 5 mg/mL MTT (dissolved in PBS) to each well, incubating for 3 h at 37 °C, then adding 100 μL DMSO, mixing, and measuring absorbance at 570 nm after 15 min.

### 4.5. Oxidative Stress and Reactive Oxygen Species

The DCFDA H2DCFDA cellular ROS assay (Abcam, ab113851) using flow cytometry determined cellular ROS levels in live cells. Cells were seeded in 12-well plates and treated with the test and control agents described in Section 4.1: Carboplatin (100 μM) and Thymoquinone (20 μM). After a 36 h incubation in normoxic and hypoxic conditions, as described in Section 4.1. Cells were detached using PBS + 4 mM EDTA and collected in suspension in 1 mL PBS. 1 × 10^6^ cells are stained with 20 μM DCFDA and incubated for 30 mins at 37 °C. Cells were analyzed on a flow cytometer; cells were gated using forward scatter and side scatter parameters. Excitation of DCF occurs at 488 nm and emission at 535 nm (FL1).

### 4.6. Apoptosis

Annexin V FITC and PI staining was used to identify early apoptotic (Annexin +, PI-), late apoptotic (Annexin +, PI+), and necrotic cells (Annexin -, PI+) using flow cytometry and the Annexin V-FITC/PI Apoptosis Kit (E-CK-A211, Elabscience, Wuhan, China). Cells were seeded in 12-well plates and treated with the test and control agents: Carboplatin (100 μM) and Thymoquinone (20 μM) after a 36 hour incubation in normoxic and hypoxic conditions, as described in Section 4.1. Cells were detached using PBS + 4 mM EDTA, washed 3× in PBS, and resuspended in 1 mL PBS. 1 × 10^6^ cells were collected from each treatment group and centrifuged to form a cell pellet. The cell pellet was resuspended in 500 μL 1× Annexin V binding working solution, and then 5 μL Annexin V-FITC and 5 μL PI were added to each tube. The cells were mixed with gentle vortexing and incubated at RT for 150–20 min in the dark. The cells were analyzed by flow cytometry. Excitation 488 nm and emission 530 nm for FITC (FL1). Excitation 535 nm and emission 617 nm for PI (FL2).

### 4.7. Confocal Imaging LSM

Cells were seeded at 5 × 10^6^ cells per well in a 6-well plate and grown overnight. Cells were treated at the IC50 concentration for 48 h in normoxic and hypoxic conditions with test and control agents, as described in Section 4.1. The cell pellets are collected and washed in PBS and resuspended in Hanks Balanced Salt Solution (HBSS), then stained with 2.5 µg/mL YO-PRO™-1 Iodide (green; excitation 491 nm and emission 509 nm; apoptotic cells), 2.5 µg/mL HOECHST33342 (blue; excitation 352 nm and emission 454 nm; live cells) and 2.5 µg/mL PI (red; 490 nm/640 nm; dead cells) for 45 min at in a humidified cell culture incubator. Images were acquired on the Zeiss LSM780 microscope system (Zeiss Group, Oberkochen, Germany).

### 4.8. Autophagy

Cells were treated with test or control agents in 6-well plates, as described in Section 4.1. An additional positive control treatment was performed, in which cells were treated with 25 mM rapamycin. Cells were incubated for 24 h during treatment in normoxic and hypoxic conditions, as described in Section 4.1. The cells were harvested using a PBS solution containing 4 mM EDTA and washed in media, then washed three times in PBS. Cell pellets were collected in a centrifuge tube and washed three times in PBS. Finally, 1 × 10^6^ cell pellets were stained with 250 μL of acridine orange (AO) or propidium iodide (PI) and incubated in the dark at 37 °C. Cells are washed again 3× in PBS. Cells were analyzed on a flow cytometer on FL1 and FL2 detectors.

### 4.9. Stem Cell Surface Marker Detection

To detect cell surface markers and phenotypic changes, expression of specific markers (CD44, CD133, and CD147) was measured using fluorophore-labeled antibodies. Antibodies used include PerCP-Cy5.5-Mouse Anti-human-CD147 IgG, FITC-Mouse Anti-human-CD44v6 IgG, and PE-Anti-human CD133 IgG. Cells are seeded in 6-well plates, treated with drugs at IC50 for 48 h under normoxic or hypoxic conditions, then detached, washed, and resuspended in PBS. 1 million cells were stained with each antibody, vortexed, incubated in the dark, and analyzed by flow cytometry in FL1, FL2, and FL3 channels.

### 4.10. Statistical Analysis

Data analysis was performed using GraphPad PRISM Software version 9.5.1 (San Diego, CA, USA). IC50 values were calculated using a non-linear regression 4-parameter log (inhibitor) vs. response variable slope. Statistical differences between basal and control, normoxic and hypoxic conditions were determined using the T-test, ANOVA, and Tukey’s multiple comparisons test. A *p*-value of less than 0.05 was considered statistically significant. All experiments were repeated at least 3 times.

## 5. Conclusions

The tumor microenvironment plays a crucial role in shaping cancer cell behavior and influencing treatment outcomes. The current findings suggest that apoptosis is a preferred mechanism, enabling the cancer to evade immune surveillance during treatment. Combining carboplatin and Thymoquinone may help reduce apoptosis and autophagy while activating alternative cell death pathways that are more effective at overcoming cancer resistance and adaptation. Despite thymoquinone’s profound cytotoxicity on the resistant phenotype, it is essential to note that plant metabolites may activate mechanisms of resistance, highlighting the dual nature of natural compounds. The current results suggest that a personalized approach to treatment is necessary, as different tumor microenvironments necessitate distinct therapeutic strategies that target both the bulk tumor and stem cell populations, and must consider the speed of cancer cell adaptation and optimal treatment kinetics. Additionally, many of these herbal compounds are absorbed into the body through the diet, which plays a significant role in shaping the tumor microenvironment and influencing the therapeutic response. Hence, further research is essential to explore the impact of the microenvironment on drugs and plant metabolites in the human body, thereby deepening our understanding of the mechanisms underlying therapeutic success and chemoresistance.

## Figures and Tables

**Table 1:** Cytotoxic potency (IC50) and efficacy (Span) of thymoquinone, carboplatin, and thymoquinone + carboplatin combined in OECM-1 cells under normoxic and hypoxic conditions.

	IC50 (µM) ± SD		Percentage Span	
	Normoxic	Hypoxic	Normoxic	Hypoxic
Thymoquinone	27.9 ± 9.4	12.0 ± 14	99.9 ± 6.1	79.5 ± 5.7
Carboplatin	81.9 ± 5.1	110 ± 15	88.0 ± 13	39.7 ± 1.4
Thymoquinone + Carboplatin	49.1 ± 33	24 ± 12	94.0 ± 9.5	91.4 ± 9.3

## Data Availability

The original contributions presented in this study are included in the article. Further inquiries can be directed to the corresponding author.
